# Supplementary material for: The genome sequence of the freshwater leech, Erpobdella octoculata (Linnaeus, 1758) (Hirudinida: Erpobdellidae)
Source: Wellcome Open Res. 2025 Nov 24;10:660. [Version 1] doi: 10.12688/wellcomeopenres.25215.1 (PMC12881851; doi:10.12688/wellcomeopenres.25215.1)
Supplement: Peer review history

# Reviewer report: Reviewer response for version 1

**DOI:** 10.21956/wellcomeopenres.27791.r144171

**Published:** 2026-02-06

**Authors:** Jun Kim

**Affiliations:** 1 Chungnam National University, Daejeon, Daejeon, South Korea

As this work was conducted by the Darwin Tree of Life Consortium, the dataset appears to be of high quality and carefully curated. The analytical workflow follows well-established standardized procedures, and potential errors seem to have been appropriately addressed.

 The only point on which I would appreciate further clarification concerns the presence of telomeric repeats. If the assemblies indeed represent complete chromosomes, one expects clusters of telomeric repeats (typically, TTAGGG or its variants) to be present at both ends of each scaffold. While it is possible that such signals may be absent due to limitations in assembly quality, it would be helpful if the authors could clarify whether telomeric repeat clusters are observed at the ends of the current chromosome-level scaffolds. Specifically, if present, it would be better to clarify how many such clusters were identified. Given a chromosome number of n = 10 in this manuscript, one might expect approximately 20 telomeric repeat clusters at the ends; it would be informative to know whether a comparable number was observed. In addition, it would be helpful to clarify whether any telomeric repeat clusters were detected internally within scaffolds (excluding interstitial telomeric sequences and considering only those located at contig or scaffold termini). This is because this internal location of telomeric repeat clusters could be a signature of scaffolding errors.

 Finally, if available, I would appreciate it if the authors could indicate whether independent experimental validation has been performed, such as karyotype analyses using DAPI staining or related approaches, and whether those results are consistent with the current inference of n = 10 chromosomes.

Are sufficient details of methods and materials provided to allow replication by others?

Yes

Is the rationale for creating the dataset(s) clearly described?

Yes

Are the datasets clearly presented in a useable and accessible format?

Yes

Are the protocols appropriate and is the work technically sound?

Yes

Reviewer Expertise:

Genomics, genome assembly, long-read sequencing, DNA damage and variant formation

I confirm that I have read this submission and believe that I have an appropriate level of expertise to confirm that it is of an acceptable scientific standard.

# Reviewer report: Reviewer response for version 1

**DOI:** 10.21956/wellcomeopenres.27791.r144170

**Published:** 2026-01-13

**Authors:** Gonghua Lin

**Affiliations:** 1 Jinggangshan University, Jiangxi, China

This study presents a chromosome-level genome of Erpobdella octoculata along with preliminary structural annotations. In recent years, many leech genomes have been elucidated, but the focus has largely been on the Hirudiniformes suborder. The genome of Erpobdella octoculata, belonging to the Erpobdelliformes suborder, contributes to understanding the evolutionary characteristics of the entire class Hirudinea and merits indexing. The authors are recommended to improve the manuscript in the following aspects:

 (1) In the Background section, the authors provide only a brief description of the research subject, which diminishes the appeal of the paper. Given the substantial number of leech genomes reported in recent years, the authors could cite relevant studies to argue for the importance of leech genome research.

 (2) For the utilization of the genome, structural annotation information is as crucial as the genome sequence itself. While the authors provide detailed descriptions of genome sequencing, assembly, quality assessment, and sequence information, the section on genome annotation is overly brief, affecting the academic and potential application value of the paper. It is recommended that the authors enhance the annotation-related content. For example, the Methods section should elaborate on how Ensembl automatic annotation was performed, including software and versions used, parameter settings, etc. The Results section should include annotation outcomes, such as length distribution, N50, and GC content of CDS. Additionally, functional annotation of these CDS could be considered, such as functional predictions by aligning with databases like NR, TrEMBL, and KEGG.

 (3) In the Genome annotation report section, the authors mention “This annotation includes 30,689 transcribed mRNAs” and “The average transcript length is 5,597.31 bp, …”. These refer to RNA-Seq data generated during the annotation process rather than annotation results.

 (4) In the Data Availability section, the authors state, “The genome will be annotated using available RNA-Seq data …”. Is this statement necessary here, or might it require rephrasing?

Are sufficient details of methods and materials provided to allow replication by others?

Partly

Is the rationale for creating the dataset(s) clearly described?

Yes

Are the datasets clearly presented in a useable and accessible format?

Yes

Are the protocols appropriate and is the work technically sound?

Yes

Reviewer Expertise:

Functional Genomics

I confirm that I have read this submission and believe that I have an appropriate level of expertise to confirm that it is of an acceptable scientific standard, however I have significant reservations, as outlined above.

# Reviewer report: Reviewer response for version 1

**DOI:** 10.21956/wellcomeopenres.27791.r140991

**Published:** 2025-12-17

**Authors:** Thomas D. Lewin

**Affiliations:** 1 Department of Biology, University of Oxford, Oxford, England, UK

**Skipp et al. 2025 R1**

 This Data Note describes the sequencing and assembly of a genome for the freshwater leech *Erpobdella octoculata*. As a standard Darwin Tree of Life report, the methods are detailed and well-described. The genome itself is of high quality: chromosome-level, with two haplotypes deposited, and good scores for the quality metrics used. Raw and assembled data is all publicly available. I have no concerns about the data or the manuscript and have only two small suggestions that the authors may wish to consider.

 First, while genome annotations by Ensembl are reported in the results, the annotation process is not described in the methods. I think at least a brief description of this process should be included in the manuscript so readers have a clear idea how this was performed and do not have to go fishing through Ensembl websites to find the information.

 Second, I think some more information is needed in the introduction about why this genome specifically could be useful. Indeed, it is a very exciting addition for a number of reasons. For instance, leech genomes are currently of interest due to a potential whole genome duplication in the lineage (refer to 1 ), something that is relatively rare in invertebrates. They also made headlines last year when three papers simultaneously reported the complete loss of macrosynteny in leeches and earthworms (refer to 2,3,4) ; and see summary in (refer to 5 ). Leech genomes have also been studied to elucidate adaptations to a blood-sucking lifestyle (refer to 6). Therefore, this genome may play a key role in the study of whole genome duplication, genome rearrangements and ecological adaptations to unusual lifestyles like hematophagy.

Are sufficient details of methods and materials provided to allow replication by others?

Yes

Is the rationale for creating the dataset(s) clearly described?

Partly

Are the datasets clearly presented in a useable and accessible format?

Yes

Are the protocols appropriate and is the work technically sound?

Yes

Reviewer Expertise:

Evolutionary Genomics

I confirm that I have read this submission and believe that I have an appropriate level of expertise to confirm that it is of an acceptable scientific standard.

## References

[1] Vargas-Chávez et al. 2025 Nat. Ecol. Evol. https://www.nature.com/articles/s41559-025-02728-1.10.1038/s41559-025-02728-140533512

[2] Vargas-Chávez et al. 2025 Nat. Ecol. Evol. https://www.nature.com/articles/s41559-025-02728-1.10.1038/s41559-025-02728-140533512

[3] Schultz et al. 2024 BioRxiv https://www.biorxiv.org/content/10.1101/2024.05.12.593736v1.full.

[4] Lewin et al. 2024 Mol. Biol. Evol. https://academic.oup.com/mbe/article/41/9/msae172/7733614.

[5] Vargas-Chávez et al. 2025 Trends. Genet. https://www.sciencedirect.com/science/article/pii/S0168952525001374.10.1016/j.tig.2025.06.00640619328

[6] Zheng et al. 2023 Gigascience. https://academic.oup.com/gigascience/article/doi/10.1093/gigascience/giad023/7113332.
